# Early Clinico-Radiographic Outcomes of Cementless Collared Triple Taper Stems in Osteoporotic Patients Undergoing Elective Total Hip Arthroplasty: A Descriptive Analysis

**DOI:** 10.1016/j.artd.2026.102109

**Published:** 2026-08-11

**Authors:** Ahmad M. Zedan, Jessica L. Abrolat, Maithily Diaz, Alan Zhong, Jeffrey J. Barry, Erik N. Hansen, Stefano A. Bini, Claudio Diaz-Ledezma

**Affiliations:** a Department of Orthopaedic Surgery, University of California, San Francisco, San Francisco, CA, USA; b UCSF School of Medicine, San Francisco, CA, USA; c UCLA, Los Angeles, CA, USA

**Keywords:** Osteoporosis, DEXA, Total hip arthroplasty, Collared triple taper stem, Radiographic outcomes, Aseptic loosening

## Abstract

**Background:**

Osteoporosis is a well-established risk factor for early femoral failure following cementless total hip arthroplasty (THA). The performance of cementless collared triple-taper stems (CTTSs) in patients with a confirmed diagnosis of osteoporosis remains poorly defined. This study aimed to evaluate early clinico-radiographic outcomes of CTTS in osteoporotic patients undergoing elective THA.

**Methods:**

We retrospectively reviewed 96 patients (87.5% women; mean age 69.5 ± 7.4 years) who underwent elective THA with a CTTS between 2018 and 2025. Osteoporosis was confirmed by either dual-energy x-ray absorptiometry T-score ≤ −2.5 or history of fragility fracture. Preoperative morphology and postoperative canal fill ratios, alignment, and subsidence (>3 mm) were assessed. Femoral failure was defined as periprosthetic fracture or aseptic loosening.

**Results:**

Preoperative morphology demonstrated a predominance of wide canals (Dorr B: 67.5%, Dorr C: 26%) and a mean morphological cortical index of 2.55. Postoperatively, high mean canal fill ratios were achieved (Z1: 0.66; Z2: 0.79; Z3: 0.76; Z4: 0.74), indicating reliable metaphyseal and diaphyseal fit. Despite osteoporotic bone quality, no cases of measurable subsidence were observed. Prominent collars occurred in 34.4% and neocortex formation in 5.2%, with no associated complications. At a mean follow-up of 2 years (range 1–7), the primary outcome of femoral failure was 0% at 90 days, 1 year, and final follow-up.

**Conclusions:**

Cementless CTTS demonstrated reliable early fixation with no femoral failures in a rigorously defined osteoporotic cohort. These findings suggest that cementless CTTS may be a viable option for femoral fixation in osteoporotic patients undergoing elective THA.

**Level of Evidence:**

Level III, Therapeutic study.

## Introduction

Osteoporosis is a significant public health challenge, affecting an estimated 16% to 26% of patients undergoing elective total hip arthroplasty (THA) [1,2]. This condition increases the risk of both surgical and medical complications after THA [3], contributes to higher readmission rates, and can compromise functional outcomes [4]. As such, osteoporosis should be recognized as an important comorbidity that requires careful assessment and management during THA planning.

Cementless stems, while widely used, have been linked to higher rates of early femoral failure than cemented stems in osteoporotic patients undergoing THA [3]. While the specific design of a cementless stem is a crucial factor [5,6], a notable gap in the literature exists regarding the performance of the newly introduced collared triple-taper stem (CTTS) in this population. Recent registry data comparing collared metaphyseal-filling cementless stems to cemented stems in patients older than 65 years of age show no discernible difference in the risk of periprosthetic fractures [7]. However, reliance on age as a surrogate for bone quality limits generalizability to patients with confirmed osteoporosis. Therefore, this data cannot be completely extrapolated to predict the performance of CTTS in an osteoporotic population.

This descriptive study aimed to evaluate the clinical and radiographic outcomes of CTTS in patients with a confirmed perioperative diagnosis of osteoporosis undergoing elective THA at an academic center. Specifically, we sought to ascertain the rates of periprosthetic femoral fracture and aseptic loosening within the initial postoperative period and to perform a radiographic analysis of stem fit and subsidence in osteoporotic bone. We hypothesized that CTTS would be associated with a low rate of early femoral failure in this high-risk cohort.

## Material and methods

### Study design and setting

We conducted a retrospective cohort study evaluating patients who underwent elective THA between January 1, 2018, and June 1, 2025. All patients included in this study were operated on by one of eight high-volume arthroplasty surgeons at a single academic medical center. The study was reported in accordance with the Strengthening the Reporting of Observational Studies in Epidemiology (STROBE) guidelines for observational studies. This study was conducted at a time point when 3 contemporary CTTS designs from multiple manufacturers had become available at our institution. This allowed evaluation across multiple stem designs within a single clinical setting.

### Participants

Patients were identified using the SlicerDicer reporting tool within the Epic electronic medical record system (Epic Systems Corporation; Verona, WI) by querying for the following procedures: “total hip arthroplasty without cement,” “total hip arthroplasty cementless,” “anterior hip arthroplasty total,” and “anterior hip arthroplasty total bilateral.” Patients were further filtered based on International Classification of Diseases (ICD)-10 diagnostic codes for osteoporosis M80.∗ and M81.∗. Each chart was individually reviewed by 4 investigators trained in orthopaedic outcomes research (A.Z., J.L., M.D., and C.D.L.). Ambiguities were resolved in consultation with the senior author (C.D.L.).

The participating surgeons also utilized cemented femoral fixation during the study period; however, the present study was designed to evaluate outcomes following cementless CTTS. All included patients received a CTTS from stem systems intended for cementless fixation, and no intraoperative conversions (“bailouts”) to cemented fixation occurred within the study cohort.

**Figure 1:**
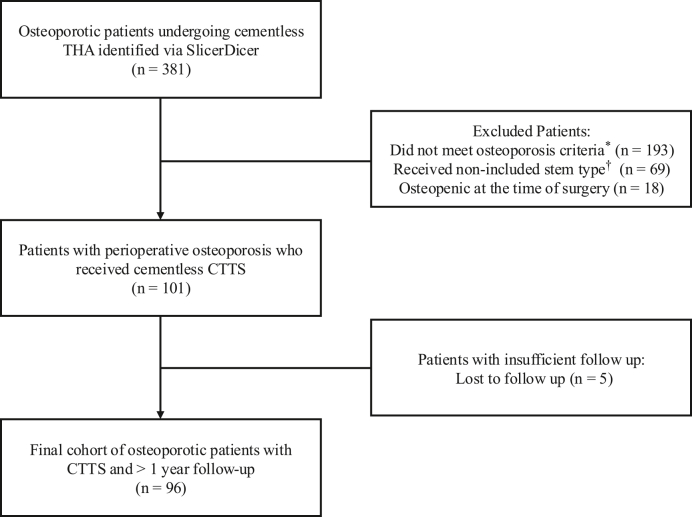
Flowchart of patient selection for study inclusion. Diagram depicts the total number of patients identified via electronic medical record (EMR) query, stepwise application of inclusion and exclusion criteria, and final cohort of 96 osteoporotic patients who underwent elective THA with cementless CTTSs. ∗Did not have DEXA T-score ≤ −2.5 or history of fragility fracture with concurrent treatment. †Received a cementless Accolade, Summit, Synergy, or Corail stem.

Patients with a formal diagnosis of osteoporosis, defined as a perioperative dual-energy x-ray absorptiometry (DEXA) scan showing a T-score ≤ −2.5, or history of fragility fracture with concurrent osteoporosis treatment at the time of surgery, were included [8,9]. Fragility fractures were identified through detailed chart review, including documented clinical diagnoses and radiographic evidence of prior low-energy fractures. Qualifying fracture sites were defined according to established osteoporosis guidelines and included hip, vertebral, distal radius, proximal humerus, pelvic, and other low-energy fragility fractures. Patients were excluded if they did not meet the diagnostic criteria for osteoporosis, had a perioperative DEXA scan consistent with osteopenia, received a femoral stem not among those specified for inclusion, received a prophylactic cable during surgery (none of the participating surgeons used prophylactic cables routinely), or had insufficient clinical follow-up of ˂1 year postoperatively. From the initial cohort of 381 patients identified via ICD-10 codes, only 96 patients met the full rigorous inclusion criteria, including both a confirmed diagnosis of osteoporosis and implantation of a CTTS, and were included in the final analysis (Fig. 1).

### Demographics and implant characteristics

A total of 96 patients (87.5% women, mean age: 69.5 ± 7.4 years [39–86]) with a mean follow-up of 2 years were included. Three cementless CTTS types were included: 69.8% Actis (DePuy Synthes; Warsaw, Indiana), 25% Insignia (Stryker; Mahwah, New Jersey), and 5.2% CATALYSTEM (Smith & Nephew; Memphis, Tennessee). Selection of the specific CTTS design was determined by the treating surgeon based on preoperative templating and surgeon preference. Both anterior and posterior surgical approaches were represented within the study cohort. Baseline and surgical characteristics stratified by sex are reported in Table 1.

**Table 1:** Baseline and surgical characteristics^a^.

Characteristic	All (n = 96)	Female (n = 84)	Male (n = 12)
Age, yr	69.5 ± 7.4	70.0 ± 7.2	66.3 ± 8.7
Height, cm	162.2 ± 8.2	160.5 ± 6.8	170.8 ± 8.5
Weight, kg	65.7 ± 13.8	63.2 ± 12.1	79.5 ± 14.2
BMI, kg/m^2^	24.7 ± 4.6	24.3 ± 4.3	27.6 ± 5.1
Surgical approach			
DAA standard table	58 (60.4%)	52 (61.9%)	6 (50.0%)
DAA Hana table	22 (22.9%)	17 (20.2%)	5 (41.7%)
Posterior	12 (12.5%)	11 (13.1%)	1 (8.3%)
Anterolateral	4 (4.2%)	4 (4.8%)	0 (0%)
Stem type			
Actis	69.8%	69.0%	75.0%
CATALYSTEM	5.2%	4.8%	8.3%
Insignia	25.0%	26.2%	16.7%
Offset			
High (HO)	54.7%	55.4%	50.0%
Standard (STD)	45.3%	44.6%	50.0%
Cup size			
46	1.0%	1.2%	0.0%
48	18.8%	21.4%	0.0%
50	26.0%	29.8%	0.0%
52	30.2%	33.3%	8.3%
54	14.6%	10.7%	41.7%
56	7.3%	2.4%	33.3%
58	1.0%	0.0%	8.3%
60	1.0%	0.0%	8.3%

DAA, direct anterior approach.

a Values are expressed as percentages unless otherwise noted.

### Osteoporosis characterization

Perioperative diagnosis of osteoporosis was supported by DEXA T-score ≤ −2.5 (87 patients, 91%, mean T-score: −3.17 [−5.7 to −2.5]) and/or history of fragility fracture with concurrent osteoporosis treatment at the time of surgery (42 patients, 43.8%) (Table 2). The average time of DEXA scans to surgery was 6.2 months, and most patients were receiving treatment for osteoporosis before their operation with 1 or more of the medication classes highlighted in Table 2.

**Table 2:** Osteoporosis characterization^a^.

Characteristic	All (n = 96)
DEXA ≤ –2.5	91%
T-score	−3.17 ± 0.63
T-score range	−5.7 to −2.5
Time from surgery (months)	6.2 ± 3.3
Fragility fracture	43.8%
Hip, n	13
Pelvis, n	2
Spine, n	9
Spine, n	5
Multiple sites	13
Treatment	
None	25%
Received before surgery^b^	21.6%
Received at the time of surgery	47.4%
Started after surgery	6%
Medication class^c^	
Bisphosphonates (orally)	69.4%
Bisphosphonates (intravenous)	9.7%
RANKLi	11.3%
PTH Analog	6.6%
PTHrP	3%

SD, standard deviation; RANKLi, receptor activator of nuclear factor kappa-B ligand inhibitor; PTH, parathyroid hormone; PTHrP, parathyroid hormone-related protein.

a Values are expressed as mean ± SD or percentages unless otherwise noted.

b Not receiving treatment at the time of surgery.

c Some patients were on multiple medications, accounting for a total > 100%.

### Radiographic assessment

The preoperative morphology of the proximal femoral medullary cavity and postoperative canal fill ratio (CFR) of the proximal femur were assessed using standardized anteroposterior (AP) pelvis radiograph. Measurements were performed by 3 independent researchers (A.Z., J.L., and M.D.). The geometric morphology of the proximal femur, including canal flare index (CFI), canal diaphysis ratio (CDR), morphological cortical index, neck-shaft angle, dysplasia defined as <25° lateral center-edge angle [10], and canal calcar ratio, were measured using preoperative radiographs, as previously described in the literature [[11], [12], [13]]. In addition, the CFI was used to classify the proximal femoral morphology into Dorr A (CFI >4.7), Dorr B (4.7 ≥ CFI ≥3.0), and Dorr C (CFI <3.0) [8,11,13,14].

**Figure 2:**
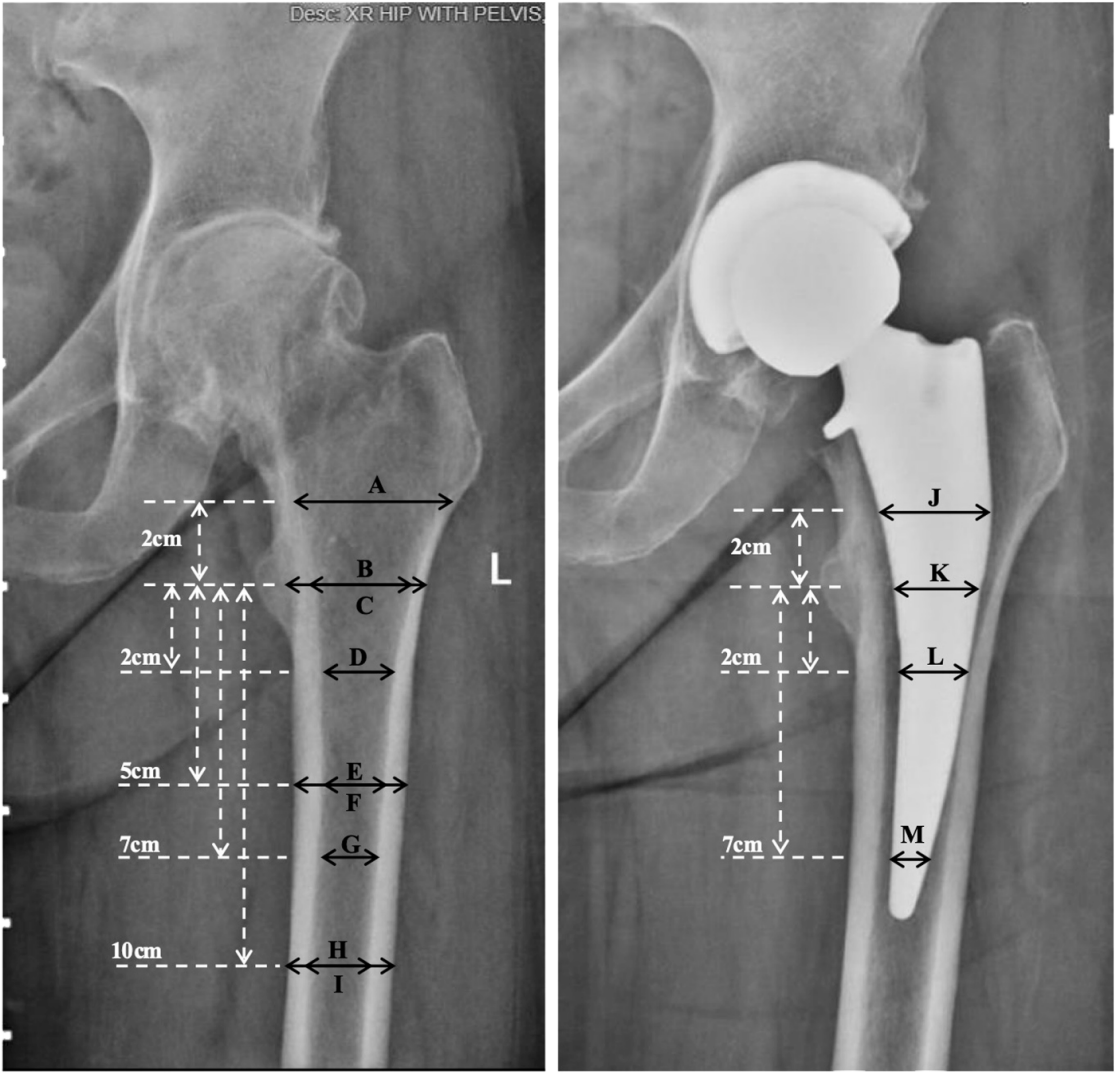
Radiographic measurements of proximal femur morphology preoperatively and stem canal fill ratio postoperatively.

**Figure 3:**
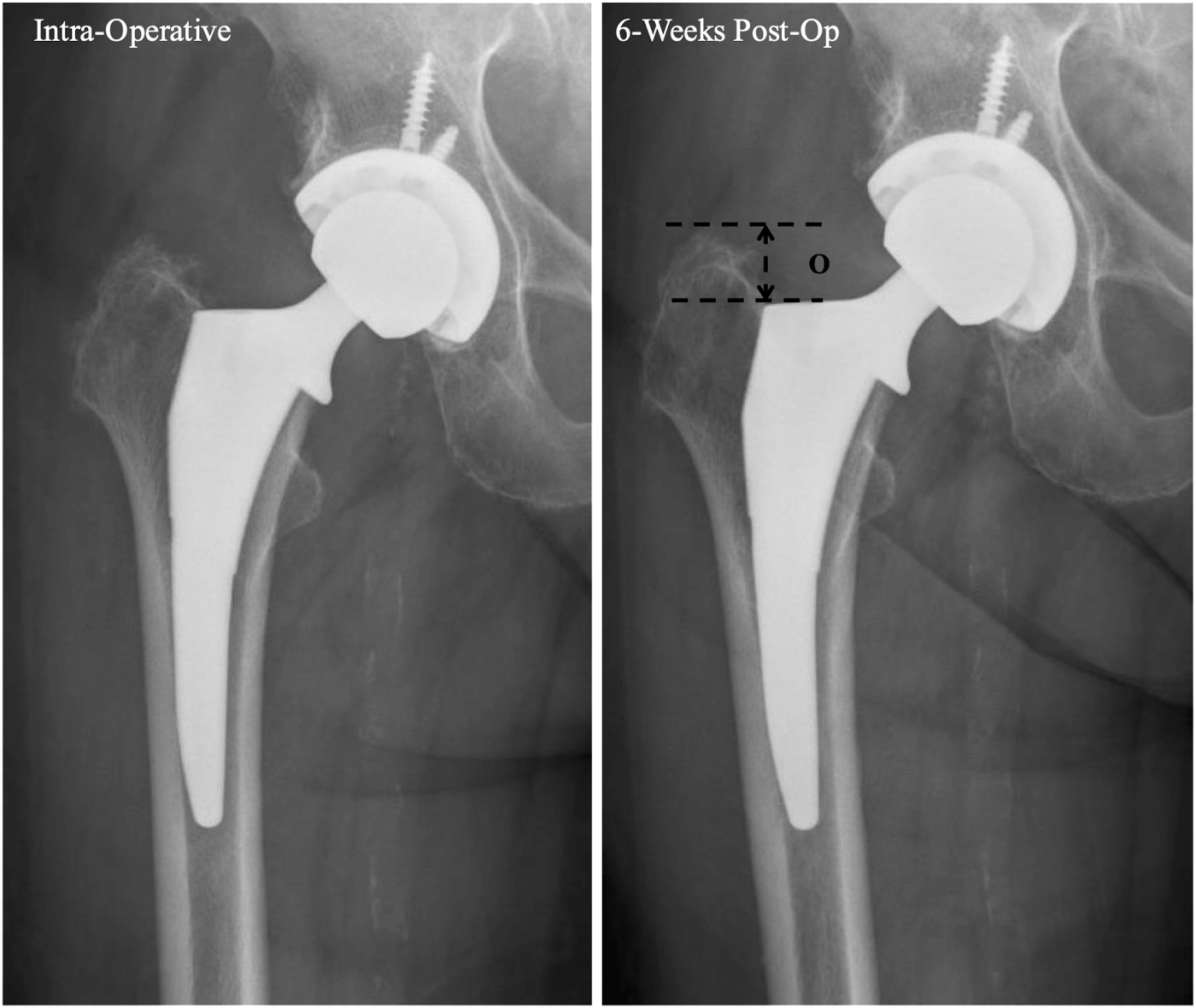
Radiographic method for measuring stem subsidence. Stem subsidence was defined as the vertical shift of the stem > 3 mm inside the femur within the first 6 weeks postoperatively. Stem subsidence was measured by assessing the distance between 2 fixed points: the tip of the greater trochanter and the flat horizontal shoulder of the prosthesis.

Postoperative AP pelvis radiographs were used to measure the CFR at 4 zones Z1, Z2, Z3, and Z4 as shown in Figure 2 and Table 3. Additionally, stem axial alignment, neocortex, and prominent or proud collars were also evaluated. Proud collars were specifically assessed because they provide a radiographic reference for detecting early stem migration and may reflect collar-calcar engagement. Subsidence was defined as >3 mm of vertical stem migration within 6 weeks postoperatively [15]. Stem subsidence was assessed in all patient cases and measured by assessing the distance between 2 fixed points: the tip of the greater trochanter and the flat horizontal shoulder of the prosthesis as shown in Figure 3 at 6 weeks and the most recent follow-up [11,15,16]. All suspected cases of stem subsidence were independently reviewed by 4 orthopaedic arthroplasty surgeons (J.B., E.H., S.B., and C.D.L.).

**Table 3:** Preoperative and postoperative radiographic measurements

Variable	Calculation
Preoperative	
Canal flare index (CFI)	A/I
Canal diaphysis ratio (CDR)	E/F
Morphological cortical index (MCI)	B/G
Canal calcar ratio (CCR)	I/C
Postoperative	
Canal fill ratio zone 1 (Z1)	J/A
Canal fill ratio zone 2 (Z2)	K/C
Canal fill ratio zone 3 (Z3)	L/D
Canal fill ratio zone 4 (Z4)	M/G

Refer to Figure 2 for measurement dimensions.

### Outcomes and Follow-up

The primary outcome was femoral failure, defined by either introperative- or postoperative periprosthetic femoral fracture or evidence of aseptic loosening. All included patients had a minimum of a 1-year follow-up, with a mean of 2 years (range 1 to 7 years). Previous literature has defined “early” femoral failure for cementless stems as occurring within the first 3 months postoperatively, and we evaluated both “early” and short-term outcomes with additional assessment at the most recent follow-up to confirm findings [17]. This timeframe is used to assess early stem performance, as prior studies suggest that differences in cementless stems’ failure rate are most pronounced early after surgery, with outcomes generally stabilizing thereafter [18].

### Data analysis

Descriptive statistics were used to summarize patient demographics, implant characteristics, and clinical outcomes. Continuous variables were reported as means with standard deviations, while categorical variables were presented as frequencies and percentages. No formal hypothesis testing or comparative statistical analyses were performed, as the study was descriptive in nature. Data were analyzed using SPSS Statistics version 29 (IBM Corp; Armonk, NY).

### IRB statement

This study was approved by the institutional review board (IRB# 20-31690) at the University of California, San Francisco, with a waiver of informed consent granted due to the retrospective nature of the analysis.

## Results

### Radiographic assessment

#### Preoperative morphology

Radiographic assessment of preoperative proximal femoral morphology revealed a mean neck-shaft angle of 129°, a mean CDR of 0.56, and a mean morphological cortical index of 2.55. Based on the CFI, Dorr type B morphology was present in 67.5% and type C in 26% of femurs. Hip dysplasia was identified in 6.3% of patients (Table 4).

**Table 4:** Preo perative radiographic femoral morphology assessment

Characteristic	Mean ± SD
Dorr class, (%)	
A	6.5%
B	67.5%
C	26%
Dysplasia, (%)	6.3%
NSA	129 ± 5.0
CDR	0.56 ± 0.06
MCI	2.55 ± 0.30
CCR	0.55 ± 0.11

CDR, morphological cortical index (MCI), neck-shaft angle (NSA), dysplasia (<25° lateral center edge angle), and CCR. CFI was used to classify the proximal femoral morphology into Dorr A (CFI >4.7), Dorr B (4.7 ≥ CFI ≥3.0), and Dorr C (CFI <3.0).

#### Postoperative evaluation

Postoperative CFR demonstrated reliable metaphyseal and diaphyseal fit across all measured levels (Z1: 0.66; Z2: 0.79; Z3: 0.76; Z4: 0.74). Stem axial alignment was neutral in the majority of cases. Neocortex formation was observed in 5.2% of cases, and prominent or proud collars were identified in 34.4%. Despite the presence of osteoporosis, no cases of measurable stem subsidence were observed at the 6-week or final follow-up (Table 5).

**Table 5:** Postoperative radiographic outcomes

Characteristic	Mean ± SD
Periprosthetic fracture, n (%)	0 (0)
Aseptic loosening, n (%)	0 (0)
Stem subsidence, n (%)	0 (0)
Canal fill ratio	
Z1	0.66 ± 0.06
Z2	0.79 ± 0.09
Z3	0.76 ± 0.08
Z4	0.74 ± 0.12
Stem axial alignment, n (%)	
0°	28.80%
1°	45.20%
2°	26.00%
Stem neocortex, n (%)	5.20%
Prominent collar, n (%)	34.40%
Proud collar, n (%)	20.80%

### Outcomes and follow-up

Among the 96 patients reviewed, no cases of intraoperative or postoperative periprosthetic femoral fracture or aseptic loosening occurred within the “early” 90-day postoperative window. At the 1-year mark and at the most recent follow-up (up to 7 years), the failure rate remained 0%, as confirmed by both clinical and radiographic outcomes (Table 5).

## Discussion

In this retrospective, short-term clinico-radiographic study, we observed no cases of femoral failure among 96 osteoporotic patients who underwent elective THA with CTTS implants from multiple manufacturers. These findings suggest that CTTS may provide a reliable fixation strategy in a population traditionally considered high risk for early femoral complications.

Our radiographic analysis demonstrated morphology consistent with osteoporosis. The mean CDR was 0.56, below the 0.59 threshold associated with osteoporosis and strongly correlated with low bone mineral density in a prior study [12]. In addition, as expected, our sample had a substantial proportion of Dorr C morphology, which is classically associated with higher risk for cementless fixation. Despite these characteristics, no early femoral failures were observed, supporting the hypothesis that CTTS design may mitigate the mechanical limitations of osteoporotic bone.

Postoperative radiographic findings support reliable fitting. CFRs were consistently high across proximal and distal zones, suggesting consistent metaphyseal and diaphyseal engagement. These values compare favorably to prior reports of other types of cementless stems in osteoporotic patients, which demonstrated lower proximal and distal fill [13]. Our findings are also aligned with a recent biomechanical study demonstrating that CTTS designs exhibit reduced subsidence, increased rotational stiffness, and greater resistance to failure in osteoporotic bone models [19].

The favorable performance observed in this cohort may be related to design characteristics shared across contemporary CTTS implants. These stems utilize a triple-taper geometry intended to optimize metaphyseal loading and proximal load transfer while reducing stress concentration within the femur. In osteoporotic bone, enhanced metaphyseal engagement and collar-calcar contact may improve initial stability and limit micromotion. Although the specific stem designs included in this study differ in surface coatings and other design features, all are intended to promote biologic fixation through osseointegration and durable proximal femoral support.

No clinically significant stem subsidence was observed in this cohort, with all cases demonstrating <3 mm of migration, below the established threshold for significance [15]. The collar is a well-recognized design feature to decrease this particular risk [20]. These findings are consistent with prior work demonstrating that modern collared, tapered stem designs maintain stability even in the setting of minimal early migration [11].

Our results contrast with previously reported femoral failure rates of 4–6% in general populations and up to 6–15% in high-risk cohorts treated with cementless stems lacking collar support or with a different tridimensional geometry [14,21]. Registry data have also demonstrated increased risk of periprosthetic fracture in patients with osteoporosis and in those receiving cementless fixation [22]. However, a recent analysis of the American Joint Replacement Registry demonstrated a decreasing trend in periprosthetic fractures over time with modern CTTSs, while another the American Joint Replacement Registry study of more than 79,000 primary THAs found no difference in revision for periprosthetic femoral fracture between collared metadiaphyseal-filling cementless stems and cemented fixation in patients aged 65 years and older [7,23]. The absence of such complications in our cohort may suggest that modern CTTS designs could possibly help mitigate these risks.

Our study has several limitations. The single-center, retrospective design introduces the potential for selection bias and limits generalizability. Although the participating surgeons performed both cementless and cemented THA during the study period, this investigation was designed specifically to evaluate outcomes following CTTS implantation in patients with confirmed osteoporosis. Approximately 100 cemented femoral stems were implanted by the same surgeon group during the study period, and therefore this cohort should not be interpreted as a consecutive series of all osteoporotic THAs performed at our institution. Most patients in this cohort had undergone osteoporosis screening and were receiving treatment, which may limit the generalizability of these findings to untreated or undiagnosed osteoporotic populations. Additionally, our sample size may produce a type II error. Nevertheless, a key strength of this study is the rigorous definition of osteoporosis. Unlike many prior studies relying on administrative data or demographic proxies, we required objective evidence of osteoporosis through DEXA or fragility fracture with active treatment, ensuring that this represents a truly high-risk population.

## Conclusions

Overall, these findings suggest that cementless CTTS may be a viable option for femoral fixation in osteoporotic patients undergoing elective THA. Further multicenter and comparative studies are needed to validate these results and to better define the role of modern designs of cementless stems vs cemented fixation in this population. As the prevalence of osteoporosis continues to rise, optimizing implant selection based on patient-specific bone quality will remain critical to improving outcomes in elective THA.

## Declaration of AI and AI-assisted technologies in the writing process

During the preparation of this work, the authors used ChatGPT (OpenAI) to assist with reviewing grammar, syntax, and sentence structure to improve clarity. After using this tool, the authors reviewed and edited the content as needed and take full responsibility for the content of the publication.

## Conflicts of interests

Claudio Diaz Ledezma serves on the editorial/governing board of the Journal of Arthroplasty and has medical/orthopaedic publication-related publications. Erik Hansen receives royalties from Corin and serves as paid consultant for Corin and Smith & Nephew. Jeff Barry serves as a paid consultant for Smith & Nephew, J&J MedTech, and Onkos Surgical. Stefano Bini, MD, receives royalties from Stryker; speaker honoraria from Zimmer Biomet; serves as a paid consultant for Zimmer Biomet; holds stock or stock option in StartUp Health and CaptureProof; receives financial/material support from Elsevier; serves on the Board of Directors of the Journal of Arthroplasty, and serves as an Elite reviewer Journal of Arthroplasty and as an Associate Editor for the Personalized Arthroplasty Society. All other authors declare no potential conflicts of interest.

For full disclosure statements, refer to https://doi.org/10.1016/j.artd.2026.102109.

## CRediT authorship contribution statement

**Ahmad M. Zedan:** Writing – review & editing, Writing – original draft, Visualization, Project administration, Methodology, Investigation, Formal analysis, Data curation, Conceptualization. **Jessica L. Abrolat:** Writing – review & editing, Writing – original draft, Visualization, Methodology, Investigation, Formal analysis, Data curation. **Maithily Diaz:** Writing – review & editing, Writing – original draft, Methodology, Investigation, Data curation. **Alan Zhong:** Methodology, Investigation, Data curation. **Jeffrey J. Barry:** Writing – review & editing, Validation, Supervision, Resources, Methodology, Conceptualization. **Erik N. Hansen:** Writing – review & editing, Validation, Supervision, Resources, Methodology, Conceptualization. **Stefano A. Bini:** Writing – review & editing, Validation, Supervision, Resources, Methodology, Conceptualization. **Claudio Diaz-Ledezma:** Writing – review & editing, Validation, Supervision, Software, Resources, Project administration, Methodology, Investigation, Formal analysis, Data curation, Conceptualization.

## Appendix A Supplementary data

Conflict of Interest Statement for Zedan

Conflict of Interest Statement for Diaz-Ledezma

Conflict of Interest Statement for Zhong

Conflict of Interest Statement for Abrolat

Conflict of Interest Statement for Hansen

Conflict of Interest Statement for Barry

Conflict of Interest Statement for Bini

Conflict of Interest Statement for Diaz
